# Use of heterologous immunoassays for quantification of serum proteins: The case of canine C-reactive protein

**DOI:** 10.1371/journal.pone.0172188

**Published:** 2017-02-21

**Authors:** Alberto Muñoz-Prieto, Asta Tvarijonaviciute, Damián Escribano, Silvia Martínez-Subiela, José J. Cerón

**Affiliations:** Interdisciplinary Laboratory of Clinical Analysis, Interlab-UMU, Regional Campus of International Excellence “Campus Mare Nostrum”, University of Murcia, Espinardo, Murcia, Spain; Duke University School of Medicine, UNITED STATES

## Abstract

The use of heterologous immunoassays containing antibodies raised against a different biological species for quantification of serum proteins is studied and discussed, taking as example the case of the use of a commercially available heterologous assay containing antibodies against human C-reactive protein (hCRP) for quantification of CRP in serum of dogs. This assay was adapted and validated for measurements of canine CRP (cCRP) and compared with three different homologous assays containing species-specific canine antibodies, which are currently commercially available for cCRP determination. Serum samples from healthy and diseased dogs (n = 44) were used. Analytical evaluation included precision, accuracy, limit of detection and lower limit of quantification for all assays. In the case of the heterologous assay also cross-reactivity of the antibody of the heterologous assay with cCRP was evaluated by a Western-Blot analysis giving a positive result. The heterologous assay showed similar results than the homologous assays in all the tests of the analytical evaluation that indicated that the assay was precise and accurate. Method comparison showed a high correlation between all assays (r≥0.9). The Bland-Altman test revealed that the heterologous assay showed a proportional error when compared with the homologous automated assays and a random error when compared with the point-of-care assay. All four CRP assays were able to detect higher CRP values in dogs with inflammatory conditions compared with healthy dogs. It is concluded that heterologous immunoassays could be used for quantification of serum proteins in different species, provided that the antibody has cross-reactivity with the protein to be measured and the assay give satisfactory results in the analytical validation tests. In addition, use of species-specific calibrators and an appropriate batch validation are recommended in these cases.

## Introduction

C-reactive protein (CRP) is a major positive acute phase protein (APP) in dogs, produced mainly in the liver in response to increased concentrations of pro-inflammatory cytokines as part of the innate immune response [1–3]. In dogs, increased CRP concentrations have been observed in a wide variety of different diseases and conditions, including infectious diseases [4,5], immune-mediated diseases [6,7] or neoplasia [8,9]. Overall CRP and in general acute phase proteins measurements are considered as the most sensitive tests to detect inflammation that can be used in clinical practice [10]. In the last years, the increasing availability of reagents and equipment for their measurements are contributing to a wider diffusion and use of this analyte.

Various types of assays have been developed for the detection of canine CRP (cCRP): immunodiffusion assays [11], time-resolved immunofluorimetric assays [12], capillary tests and slide reverse passive latex agglutination tests [13]. In addition, canine specific enzyme-linked immunosorbent assays (ELISA) have been developed and in some cases have been used as reference methods for the validation of new assays [14,15]. However, ELISA assays can have a higher imprecision due to manual pipetting, and take much time and labour to perform. Additionally, there are immunoassays that can be used for cCRP measurements in automated biochemistry analysers [16] and also in-house methods that allow prompt determination of CRP at the patient side [15]. Recently a number of homologous automated and in-house immunoassays using species specific antibodies against cCRP have been developed and are commercially available. However, some years ago heterologous assays based on antibodies against human CRP (hCRP) that have cross-reactivity with cCRP were the only option for rapid and high sample throughput measurements of cCRP [17,18].

The purpose of this work was to perform an analytical validation of a commercially available heterologous immunoassay for automated measurements of cCRP and compare it with three different homologous immunoassays. Although all these assays are currently commercially available no comparative studies between their analytical performances have been published. The heterologous immunoassay evaluated is designed for hCRP measurements (Olympus^®^). The three homologous assays were: two automated canine-specific assays (Avacta Animal Health^®^ and Gentian^®^) and a point-of-care canine-specific dry-chemistry assay for in-house CRP measurement (Fujifilm^®^). The analytical evaluation included precision, accuracy, limit of detection and lower limit of quantification. Additionally, the different assays were compared in samples from healthy dogs and dogs with inflammatory conditions. This study can be taken as a case of how an immunoassay can be validated and adapted for being used for quantification of serum proteins in a different species to that was initially developed.

## Materials and methods

### Animals and sampling procedures

A total of 44 canine serum samples from dogs of various breeds ((Mongrel (15), Boxer (5), German Shepherd (4), Labrador Retriever (3), Poodle (2), Doberman (2), Rotweiler (2), Cocker Spaniel (2), Beagle (2), Spanish greyhound (1), Schnauzer (1), Dachshund (1), Belgian Shepherd (1), Golden Retriever (1), French bulldog (1), Bull terrier (1) and sexes (24 females, 20 males)) left after the routine clinical pathology analysis performed at our laboratory, were used in this study. Eight of these samples were used for the analytical validation, and 36 for the clinical validation and comparison study, where 16 were healthy dogs and 20 adult dogs with different inflammatory conditions. These dogs had data from general clinical examination, as well as of hematological and biochemical analysis. Furthermore, diagnostic tests for diseased dogs were conducted at the discretion of the attending clinician.

In all cases samples proceeded from venous blood taken at different veterinary clinics of southern Spain into a serum tubes without anticoagulant and centrifuged at 3,500 x g for 5 min at room temperature. Owners consent was obtained for all the samples. These samples were not collected solely for this study but rather for either diagnostic purposes or for health assessment, therefore, committee ethical approval was not necessary.

### Assays procedures

The heterologous assay used in this study was the Olympus^®^ CRP, an immunoturbidimetric assay designed for determination of hCRP that uses goat anti-hCRP antibodies (lot. 7543).

The homologous assays used in this study were (1) Avacta Canine C-Reactive Protein bio-analyser assay (Avacta Animal Health^®^) that is a canine species-specific immunoturbidimetric assay (lot. 130412); (2) Gentian Canine CRP Immunoassay (Gentian^®^), a canine species-specific turbidimetric immunoassay that uses polyclonal chicken anti-canine CRP antibodies (lot. 1509401–4) and (3) Fujifilm Dri-Chem Slide vcCRP, a canine-specific dry chemistry assay that uses a monoclonal mouse anti-canine CRP antibody (lot. 104408; Fujifilm^®^). No information about the source of the antibodies was provided in Avacta^®^ kits.

All immunoturbidimetric assays were run on an automated analyser (Olympus AU600, Olympus Diagnostica GmbH^®^, Hamburg,Germany). The Fujifilm^®^ assay was run at the equipment Fuji Dri-Chem NX500 (Fujifilm Corporation^®^, Tokyo, Japan) according to the manufacturers recommendations. All assays were calibrated with the test-specific calibration material provided by the manufacturer, with the exception of the Olympus^®^ assay. In this assay, instead using the calibrator provided by the manufacturer, cCRP purified by affinity chromatography following a previously published protocol [12] was used.

In the Fujifilm^®^ assay, the analyser diluted the samples 21 times automatically and for the Avacta assay a manual predilution 1:30 of the samples was made as recommended by the manufacturer.

### Method validation

The intra-assay variation was expressed as the coefficient of variation (CV) and was determined by measuring three canine serum samples, with high (>100 mg/L), medium (≈50 mg/L) and low (<10 mg/L) CRP concentrations, 5 times in the same analytical series. The inter-assay variation was assessed measuring the same samples on 5 different days.

For accuracy assessment purified cCRP was used as control material and its value was determined by the mean of the values obtained in five measurements of the total protein content of this material [19].

Accuracy was evaluated by various methods:

- By the difference between the mean of 5 measurements of the control material made with each different assay and the mean value of the control material measured by the Bradford assay.
- By a recovery study in which a serum sample with a low CRP concentration was spiked with purified cCRP to predicted three different CRP concentrations (77, 44 and 10 mg/L). Samples in the recovery study were measured in triplicate in a single run.
- By linearity under dilution study by using serial dilution of two canine serum pools with known high CRP concentrations that were measured in all methods. The samples were serially diluted with ultrapure water in case of immunoturbidimetric assays and a specific diluent provided by the manufacturer in the dry-chem method to achieve 6 concentrations (undiluted; 1:2; 1:4; 1:8; 1:16; 1:32).

The detection limit was assessed by the lowest concentration of CRP that could be distinguished from a specimen of zero value in each assay based on data from 12 replicate determinations of the zero standard (ultrapure water in immunoturbidimetric assays and assay buffer in the dry-chem method) as mean value plus two standard deviations [20].

The lower limit of quantification was calculated based on the lowest CRP concentration that could be measured with intra-assay CV<20% [20].

### Cross-reactivity for heterologous assay

A Western-Blot analysis was performed to evaluate the cross-reactivity of the heterologous assay. One human serum sample, one dog serum sample and purified cCRP were analysed. The human serum was from clotted blood obtained by venepuncture from a patient with high CRP concentration. The collection procedure was approved by the University of Murcia ethics committee and previous informed oral consent was obtained from the patient.

Human and canine serum samples were diluted 1:50 with ultrapure water and when denatured, 1,4-Dithiotrheiol was used. After, proteins were separated by sodium dodecyl sulfate polyacrylamide gel electrophoresis (SDS-PAGE) and electrophoretically transferred to a nitrocellulose membrane, which was blocked with Roti-block blocking solution overnight. Western blotting was performed using the goat anti-hCRP antiserum (Olympus^®^, dilution 1:500) and rabbit anti-goat-IgG as secondary antibody.

### Assay comparison and values in dogs with inflammatory conditions

Twenty canine serum samples which suffered from different inflammatory diseases producing increases in CRP concentrations (11 from dogs with canine leishmaniosis and 9 with pyometra) and also 16 serum samples from healthy dogs that were analysed for routine check-ups and that showed no changes at physical examination or other analytical tests and that had CRP values inside our laboratory reference range, were selected from our database.

These samples were taken at different veterinary clinics of southern Spain and were used for the comparisons of the assays and to study the ability of the evaluated assays to differentiate between healthy and diseased animals.

### Statistical analysis

Intra and inter-assay CVs and lower limits of detection and quantification (Lower and upper) were calculated using routine descriptive statistical procedures with IBM SPSS (Version 21, Enhingen, Germany). The coefficients of variation (CV) of the assays were calculated as the standard deviation (SD) divided by the mean value of analysed replicates x 100%. The lower limits of quantification and linearity under dilution were calculated using Excel 2010 (Microsoft Corp., Redmond, WA).

Data showed a non-parametric distribution through Kolmogorov-Smirnov test. Therefore, Spearman test was performed to assess the correlation between the different methods, Wilcoxon test for paired samples was selected to investigate the differences between assays in the groups of normal and high CRP values and Mann-Whitney test was used to compare the ability of each assay to differentiate between samples with normal and high CRP values. In the method comparison study, a Bland-Altman plot including the 95% limits of agreement was set to evaluate the difference between methods [21].

## Results

### Method validation

As shown in Table 1, intra-assay CVs showed minimum and maximum values of, 1.12–6.13%, 1.28–8.86% 0.96–5.84% and 2.01–7.85% for the Olympus^®^, Avacta^®^, Gentian^®^ and Fujifilm^®^ assays. Inter-assay precision showed a minimum and maximum values of 2.46–9.94%, 8.10–12.86%, 4.08–6.05% and 3.55–14.41% for Olympus^®^, Avacta^®^, Gentian^®^ and Fujifilm^®^ assays (Table 2).

**Table 1:** Intra-assay repeatability of the assays for detection of a high (>100 mg/L), medium (≈50 mg/L) and low (≈10 mg/L) concentrations of canine C-reactive protein.

Test	High	Medium	Low
	(>100 mg/L)	(≈50 mg/L)	(≈10 mg/L)
Olympus			
Mean ± SD	105.64±1.19	64.22±3.94	10.04±0.58
CV (%)	1.12	6.13	5.77
Avacta			
Mean ± SD	111.93±1.82	33.44±0.43	4.40±0.39
CV (%)	1.62	1.28	8.86
Gentian			
Mean ± SD	104.42±1	52.88±0.69	6.84±0.40
CV (%)	0.96	1.31	5.84
Fujifilm			
Mean ± SD	167.4±3.36	56.2±1.48	11.4±0.89
CV (%)	2.01	2.64	7.85

Mean, SD and CV were calculated from 10 replicate measurements of the different concentrations.

**Table 2:** Inter-assay repeatability of the assays for detection of a high (>100 mg/L), medium (≈50 mg/L) and low (≈10 mg/L) concentrations of canine C-reactive protein.

Test	High	Medium	Low
	(>100 mg/L)	(≈50 mg/L)	(≈10 mg/L)
Olympus			
Mean ± SD	107.1±3.17	70.4±12.5	5.5±0.54
CV (%)	2.96	2.46	9.94
Avacta			
Mean ± SD	97.09±12.49	28.67±3.14	7.8±0.6
CV (%)	12.86	10.94	8.1
Gentian			
Mean ± SD	109.88±4.48	56.60±2.66	3.18±0.19
CV (%)	4.08	4.70	6.05
Fujifilm			
Mean ± SD	150.8±14.25	57.8±2.05	12.2±1.29
CV (%)	9.45	3.55	14.41

Mean, SD and CV were calculated from 5 replicate measurements on 5 different days.

Measurements of the control material consisting in pure cCRP having a mean value of 42 mg/L showed an inaccuracy of 2.2, 4.5, 3.8 and 9.8% for the Olympus, Avacta, Gentian and Fujifilm assays, respectively.

The spiking recovery study showed that the difference between observed and expected CRP concentrations varies from 103 to 118%, 111 to 117%, 105 to 118% and 87 to 116% for Olympus, Avacta, Gentian and Fujifilm assays, respectively.

**Fig 1:**
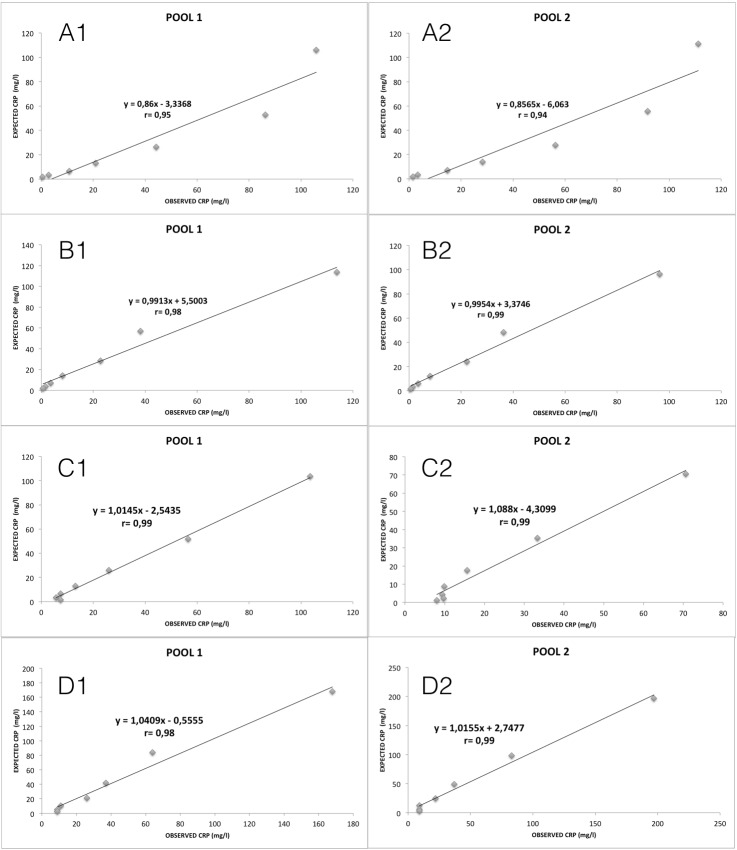
Linearity range of the assays for canine CRP determination. A1: Olympus with sample 1; A2: Olympus with sample 2. B1: Avacta with sample 1; B2: Avacta with sample 2. C1: Gentian with sample 1; C2: Gentian with sample 2. D1: Fujifilm with sample 1 D2: Fujifilm with sample 2.

The results obtained from the dilution of two pools with known concentration of CRP showed in all cases linear regression equations with coefficient of correlation closed to 1 (Fig 1).

The analytical limits of detection were 0.97, 0, 0 and 3 mg/L for Olympus, Avacta, Gentian and Fujifilm CRP assays, respectively. The lower limits of quantification were 2.82, 1.39, 5.4 and 10.4 mg/L for Olympus, Avacta, Gentian and Fujifilm assay, respectively.

### Cross-reactivity of the heterologous assay

**Fig 2:**
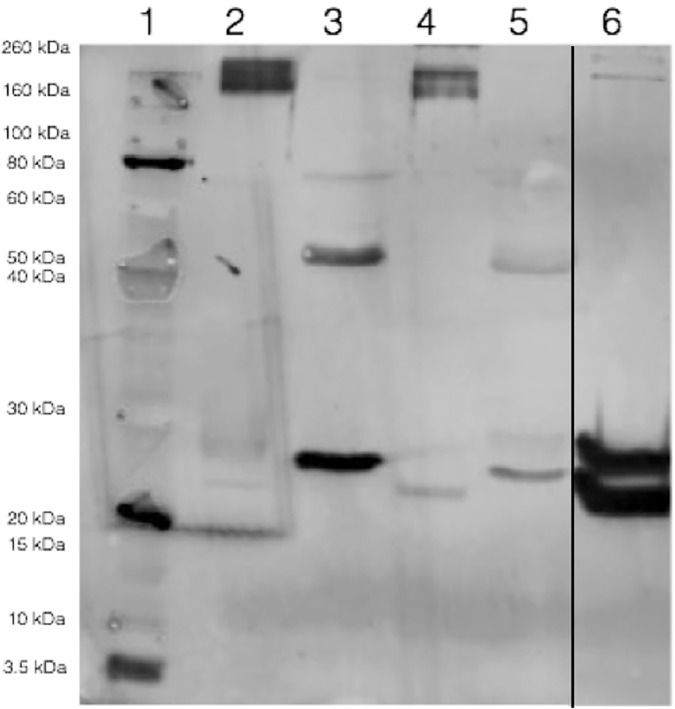
Qualitative detection of canine C-reactive protein (CRP) by Western blotting analysis. Lane 1: protein standard; Lane 2: human serum exposed to non denatured conditions; Lane 3: human serum exposed to denatured conditions; Lane 4: dog serum exposed to non denatured conditions; Lane 5: dog serum exposed to denatured conditions; Lane 6: purified canine CRP.

The antibody of the heterologous assay showed cross-reactivity with purified cCRP, human and dog serum samples by Western-Blot analysis (Fig 2). Two bands of approximately 24 y 28 kDa were observed when purified cCRP was analysed (lane 6). Similar bands could be identified with human and canine serum, which represent the different multimeric forms of the CRP molecule.

### Assay comparison and values in inflammatory conditions

**Fig 3:**
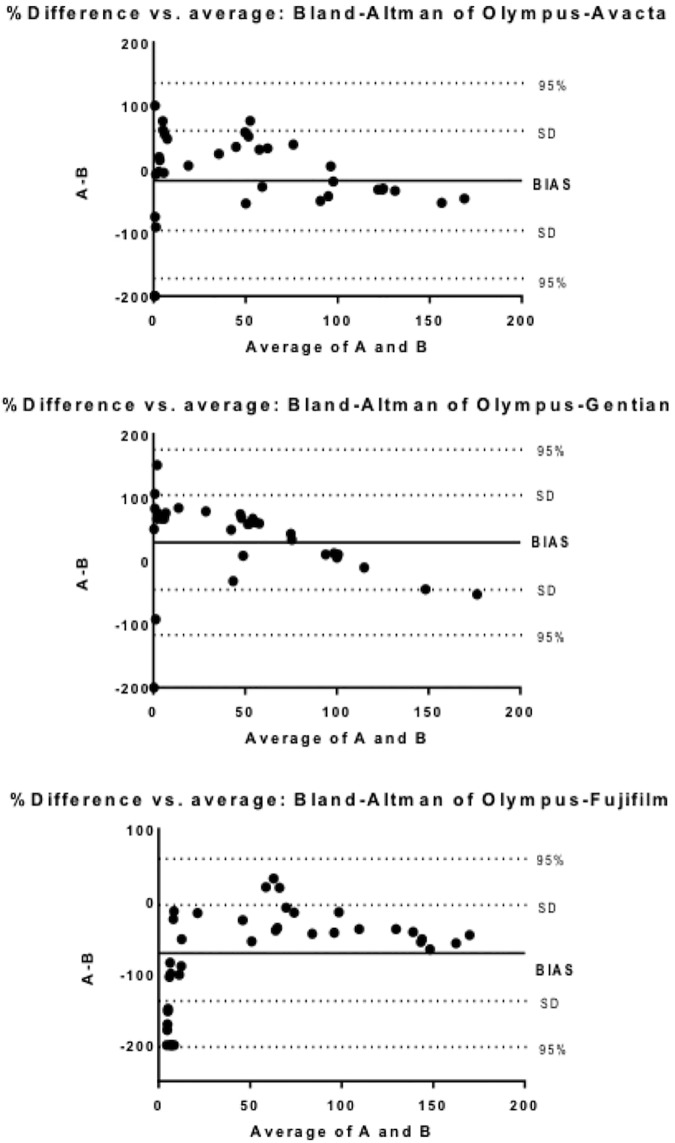
Bland-Altman difference plot for C-reactive protein (CRP) with four assays.

All methods showed a high correlation between them with the Spearman test (R≥0.9) (Table 3). The Bland-Altman test revealed that the heterologous assay showed a proportional error when compared with the homologous automated assays and a random error when compared with the point-of-care assay (Fig 3).

**Table 3:** Coefficient of Spearman correlation between the heterologous assay and the homologous assays (n = 36).

Test	Fujifilm	Avacta	Gentian
Olympus			
R	0.90	0.96	0.96
p-value	<0.01	<0.01	<0.01

**Fig 4:**
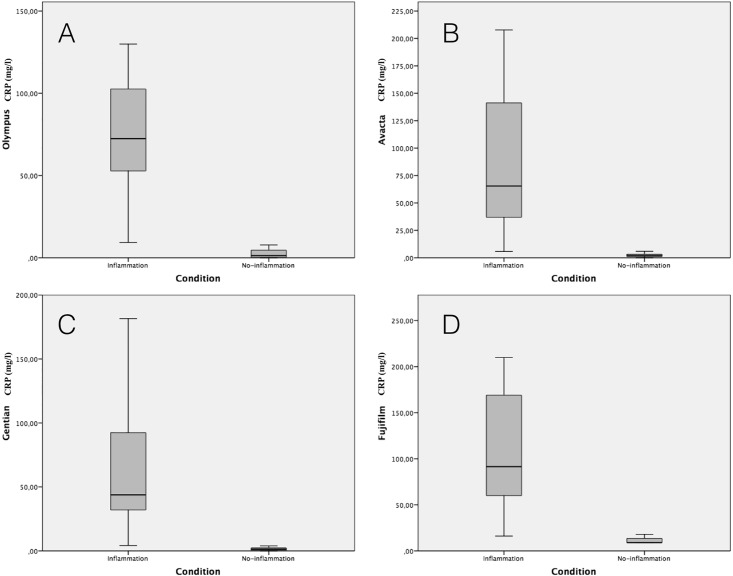
C-reactive protein concentrations in dogs with and without inflammation with the different assays. The plot shows median (line within box), 25^th^ and 75^th^ percentiles (box), 5th and 95th percentiles (whiskers). p value is indicate when significant difference between assays exists.

**Fig 5:**
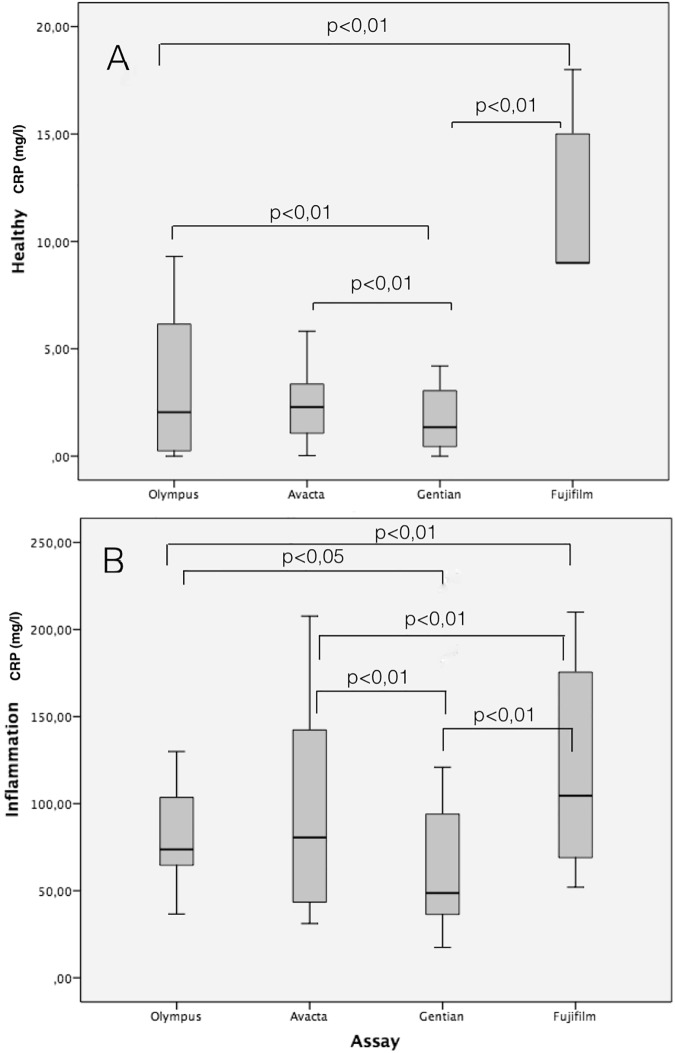
Differences between assays in dogs with (n = 20) and without inflammation (n = 16). The plot shows median (line within box), 25th and 75th percentiles (box), 5th and 95th percentiles (whiskers). P value indicates statistically significant difference between assays.

Values of the four CRP assays in the healthy and diseased dogs are presented in Table 4. All four CRP assays were able to detect higher CRP values in dogs with inflammatory conditions compared with healthy dogs and showed significant differences between the two groups (Fig 4). Significant differences between the different methods in each of the groups were observed. In healthy dogs, the heterologous assay showed similar values to those of one the homologous assays (Avacta), but significant lower and higher values when compared with the other homologous assays (lower in the case of Fuji and higher in the case of Gentian). Similar findings were found in the samples from dogs with inflammatory conditions (Fig 5).

**Table 4:** Median and range of the C-reactive protein concentrations in healthy dogs, and dogs with inflammation.

Condition	CRP (mg/L), median (range)			
	Olympus	Avacta	Gentian	Fujifilm
Healthy (n = 16)	2.82	2.29	1.35	10.4
	(0–9.3)	(0.03–5.82)	(0–4.2)	(10.4–18)
Inflammation (n = 20)	73.7	80.5	48.6	104.5
	(36.6–105.9)	(37.23–114.6)	(17.4–97.5)	(52–198)

## Discussion

In this study a heterologous method for cCRP quantification was validated and its results compared with the validation of other three different homologous assays. Overall, the results of the analytical validation showed that the heterologous assay was able to measure cCRP, showing similar results at the different validation tests than the others homologous assays. Therefore, all assays showed imprecision values lower than 20% which is considered as the acceptable limit [22]. When the accuracy was investigated by measuring purified CRP, all the assays showed inaccuracies lower than 15% and therefore are able to properly quantify cCRP [22]. Ideally a certified reference material should have been used to test the accuracy, however, the use of purified CRP could be an alternative way to assess the accuracy of a test where no available standard material exists, as in case of cCRP assays. The spiking recovery test in all cases showed values within the acceptance range for the recovery studies of 80–120% [23], is indicating that the evaluated assays can also accurately measure purified CRP when added to serum, and therefore excluding an evident matrix effect of the serum. Linearity under dilution tests additionally indicated that the assays could also detect and accurately measure different CRP concentrations. It should be pointed out that the lower regression value that showed the Olympus assay with the pool 2 was due to a prozone effect that this method showed with samples with CRP higher than 100 mg/dl, for these cases the samples should be diluted and further analysed. This prozone effect was not observed for any homologous assay even when samples of very high CRP values (400 mg/dl) were analysed (data not shown).

Method comparison showed a high coefficient of correlation between the heterologous and homologous assays. However, the values were in all cases below 0.96, and therefore a possible systematic error could occur [24] that was observed in the Bland-Altman plot. All assays showed limits of quantification lower than 5.4 mg/L, with the exception of the “in-house” assay that showed a limit of quantification of 10.4 mg/L. The upper limits of the reference ranges of CRP in healthy dogs described in literature (<10 mg/L [25], <16.4 [26] and <20 mg/L [27]) are usually higher than the limits of quantification of the assays of our study. Therefore, for interpretative purposes it could be considered that samples with CRP values lower than the limit of quantification of the assays, although are not precise, are in the reference range of healthy dogs.

All the assays were able to detect a significant increase in median CRP values in dogs with inflammatory diseases. However, the magnitude of increase between healthy and dogs with inflammation was different between assays. These data would indicate the need of establishment of particular reference ranges and cut-offs points for CRP depending on the method used for CRP measurement.

In our laboratory we are using the automated heterologous assay of this study for years, paying special attention to 2 points. One is the calibrator material used for cCRP measurements, since it is strongly recommended to use cCRP as calibration material, especially if antibodies against hCRP are used as reagents. Use of hCRP as calibrator produces lower differences than expected between healthy dogs and dogs with inflammation, and although there are reports published that use this calibrator [28], this procedure should be considered as no optimal for cCRP measurements. When the absorbance value of the reaction between cCRP and the human antibody (with a no complete cross-reactivity) are extrapolated to values of a reaction between hCRP and human antibody (with complete cross-reactivity) [29], it results in an underestimation of the values of the cCRP reaction. The use of cCRP as calibrator provide similar conditions of the reaction that occurs with canine serum, and therefore opens the possibility to obtain true values of cCRP with a partial cross-reactivity of the antibody used in the reaction.

Another important point is to carefully evaluate the antibodies used in the assay. In our case Western blotting confirmed that the antibody recognizes purified cCRP that showed a double band pattern similar to that previously described in dogs (two bands of around 24 and 28 kDa). This double pattern is due to glycosylation in two of the five subunits in each CRP molecule [12]. In addition, our results showed that the antibody recognized cCRP of serum in both pentameric full length (157 kDa) [11] and monomeric forms (28 and 24 kDa). It must be pointed out how important it is to do this type of studies when working with heterologous assays to evaluate if the antibody is able to recognize the target protein. Although some human assays have been proven to have cross-reactivity with cCRP [28,18] others were not able to measure this protein [30]. In addition it is required to evaluate the possible variations due to changes in the antibody batch produced. In our laboratory we have been measuring CRP in routine clinical activity with the human Olympus assay for more than ten years. During this time we have performed validation of each different batch of the reagent by evaluating precision, accuracy using purified cCRP, lower limit detection and method comparison between new and the previous validated batch. Meanwhile, we have not observed major differences between the different batches that could affect our clinical decision levels; the only variations observed were in the range of linearity of the assay (for example, the lower limit of detection of the assay ranged between 2 and 5 mg/L in the different batches). However, batches with insufficient cross-reactivity have been found in other heterologous assays [29].

The data provided in this paper should be interpreted with caution since it has been obtained with an specific automated analyser and therefore it could vary depending on the analyser used, and as previously mentioned, variations in the results can occur depending on the batch of antibody used in each assay. However overall, it can be concluded that use of heterologous assays with cross-reactivity with cCRP, that give satisfactory results in an analytical validation trial and calibrated with purified cCRP still constitutes an alternative for CRP measurements in dogs. In the past, the use of hCRP assays was the only option for automated measurements of cCRP, situation that can be currently extrapolated to other serum proteins in different biological species, where only immunoassays designed for a different species are available. In addition, use of heterologous assays can have a suitable alternative in situations where the available commercial species-specific tests are more difficult to obtain or expensive.
